# Thymic Atrophy and Apoptosis of CD4^+^CD8^+^ Thymocytes in the Cuprizone Model of Multiple Sclerosis

**DOI:** 10.1371/journal.pone.0129217

**Published:** 2015-06-08

**Authors:** Izabella Solti, Krisztian Kvell, Gergely Talaber, Sara Veto, Peter Acs, Ferenc Gallyas, Zsolt Illes, Katalin Fekete, Petra Zalan, Arpad Szanto, Zita Bognar

**Affiliations:** 1 Department of Biochemistry and Medical Chemistry, University of Pecs, Pecs, Hungary; 2 Department of Pharmaceutical Biotechnology, University of Pecs, Pecs, Hungary; 3 Karolinska Institutet, Department of Biosciences and Nutrition, NOVUM, Huddinge, Sweden; 4 Department of Neurology, University of Pecs, Pecs, Hungary; 5 MTA-PTE Nuclear-Mitochondrial Research Group, Pecs, Hungary; 6 Szentagothai Research Center, University of Pecs, Pecs, Hungary; 7 Department of Neurology, Odense University Hospital, Institute of Clinical Research, University of Southern Denmark, Odense, Denmark; 8 Department of Urology, University of Pecs, Pecs, Hungary; Wayne State University, UNITED STATES

## Abstract

Previous studies on the degenerative animal model of multiple sclerosis suggested that the copper-chelator cuprizone might directly suppress T-cell functions. Peripheral T-cell function in the cuprizone model has already been explored; therefore, in the present study, we investigated, for the first time, how cuprizone feeding affects the thymus, the organ of T-cell maturation and selection. We found that even one week of cuprizone treatment induced significant thymic atrophy, affecting the cortex over the medulla. Fluorescent microscopy and flow-cytometric analyses of thymi from cuprizone- and vehicle-treated mice indicated that eradication of the cluster of the differentiation-4 (CD4)-CD8 double-positive T-cell subset was behind the substantial cell loss. This result was confirmed with CD3-CD4-CD8 triple-staining experiments. Ultrastructurally, we observed degraded as well as enlarged mitochondria, myelin-bodies, large lipid droplets, and large lysosomes in the thymi of cuprizone-treated mice. Some of these features were similar to those in physiological and steroid-induced accelerated aging. According to our results, apoptosis was mainly of mitochondrial origin mediated by both caspase-3- and apoptosis inducing factor-mediated mechanisms. Additionally, mitogen activated protein kinase activation and increased pro-apoptotic B cell lymphoma-2 family protein expression were the major underlying processes. Our results do not indicate a functional relationship between cuprizone-induced thymus involution and the absence of inflammatory responses or the selective demyelination observed in the cuprizone model. On the other hand, due to the reversible nature of cuprizone’s deleterious effects, the cuprizone model could be valuable in studying thymus regeneration as well as remyelination processes.

## Introduction

Administration of the copper chelator cuprizone to young adult C57BL/6 mice induces multi-focal demyelination mainly in the corpus callosum and superior cerebellar peduncle [1] without significant T-cell activation in the affected areas [2]. According to histopathological studies [3], the pattern of cuprizone-induced demyelination resembles that of type III multiple sclerosis (MS) lesions characterised by oligodendrocyte degeneration and minor inflammation [4]. Therefore, the cuprizone model was extensively used for studying the degenerative aspects of MS [5]. The mechanism of the oligodendrocyte loss and demyelination in the cuprizone model is not well understood. Mature oligodendrocytes seem to be the primary targets, which are eliminated by apoptosis inducing factor (AIF)-mediated apoptosis [6]. Cuprizone-induced early formation of mega-mitoachondria in the liver [7] and oligodendrocytes [8], and expressional and functional changes of mitochondrial enzymes [9,10] indicate mitochondrial dysfunction behind the oligodendrocyte loss. However, there is no explanation for the preferential regional distribution of cuprizone-induced demyelination, and the exclusivity of the cell death toward oligodendrocytes.

Unlike the cuprizone model, experimental allergic encephalomyelitis (EAE) reflects the autoimmune feature of MS [11,12]. In this model, after immunising the animals with myelin antigens, myelin-specific CD4^+^ T-cells are initially activated in the peripheral immune organs and migrate to the central nervous system (CNS) [13] where they encounter their cognate antigen on CNS antigen presenting cells and produce immune mediators such as pro-inflammatory cytokines and chemokines. These immune mediators locally activate the second cascade of the autoimmune response involving microglia, the resident macrophage [14,15]. In contrast, demyelinating areas in the cuprizone model, were reported to lack B and T-cells, and the blood brain barrier was found to be intact [2,16]. Additionally, with respect to the cuprizone model, RAG-1(1/1) mice, which lack mature B and T lymphocytes are indistinguishable from controls, indicating that T-cells may not play a role in cuprizone-induced demyelination [16]. The major difference between type III MS lesions and cuprizone-induced demyelination is the presence of perivascular inflammation and CD3^+^ T-cells in the former [4]. This difference, along with the down-regulation of EAE by cuprizone-treatment [16–18] suggests that cuprizone may directly suppress T-cell functions [19]. A previous study [17] has already explored peripheral T-cell function in the cuprizone model. However there is no data regarding the thymus, the organ of T-cell maturation and selection. To elaborate on the suggested suppressive effect of cuprizone on T-cell function, in the present study, we investigated how cuprizone feeding affects the thymus.

## Materials and Methods

### Ethics Statement

The investigation conforms to the Guide for the Care and Use of Laboratory Animals published by the U.S. National Institutes of Health (NIH Publication No. 85–23, revised 1996), and was approved by the Animal Research Review Committee of the University of Pecs, Medical School.

### Animals and cuprizone administration

C57BL/6 male mice were purchased from Charles River Laboratories Hungary Ltd (Isaszeg, Hungary) and kept under standardised, specific pathogen free circumstances. Starting at four weeks of age, mice received a diet of powdered rodent chow containing 0.2% cuprizone (bis-cyclohexanone oxaldihydrazone) (Sigma, Steinheim, Germany) by weight for three or seven days *ad libitum*. Age and gender matched littermates receiving powdered rodent chow served as the control group.

### Acquisition of thymus samples

The weight of the mice was measured at the beginning and at the end of the treatment period. Then, animals were euthanised with an overdose of ketamine hydrochloride intraperitoneally, and their chest was opened. The thymi were photographed, carefully dissected and their wet weight was measured. They were freshly processed for RNA isolation, fixed for electron microscopy, frozen for histochemistry and immunohistochemistry, or homogenised in phosphate buffered saline (PBS) with a glass/glass homogeniser. Alternatively, thymic cell suspension was prepared by gentle mechanic agitation followed by filtering through a nylon mesh. The suspension was washed once and the cells were resuspended in PBS. An aliquot of cells was diluted 1:100, the cells were counted using the Trypan blue method with a hemocytometer, and the cell number was set to 5x10^5^ or 10^6^ per sample.

### Thymic epithelial cell (TEC) enrichment

TECs were enriched as previously described [20]. Briefly, the thymic lobes were digested using a collagenase-based solution. Then TECs were labelled with anti-epithelial cell adhesion molecule- (EpCAM1) antibody followed by their direct enrichment using magnetic beads (Dynabeads). TEC purity was repeatedly found to be approx. 90% based on qRT-PCR measurements as published previously.

### Apoptosis detection

For apoptosis detection, double staining with Annexin V-fluorescein isothyocyanate (FITC; BD Pharmingen, CA) and propidium-iodide (Sigma-Aldrich) was performed according to Vermes et al. [21]. Briefly, 5x10^5^ thymocytes were resuspended in 100 μl Annexin binding buffer (10mM HEPES/NaOH, pH 7.4, 140mM NaCl and 2.5mM CaCl_2_) and incubated for 20 minutes at room temperature with Annexin V-FITC in the dark, before then being diluted with 400 μl Annexin binding puffer. Propidium-iodide was given to the cells immediately before the flow-cytometric analysis. Two-parameter dot-plots showing Annexin V/propidium-iodide staining (FL1/FL3 channels) were created to determine the ratio of apoptotic cells in the thymus glands.

### Flow-cytometry

For the simultaneous detection of cell surface expressed CD4, CD8 and CD3, a triple labelling technique was used. Thymocyte samples were incubated with monoclonal antibody cocktails for 30 min in 100 ml binding buffer on ice (PBS containing 0.1% NaN_3_ and 0.1% BSA), then washed twice in PBS, and finally resuspended in 500 ml 0.1% buffered PFA (paraformaldehyde) in PBS. For staining the following monoclonal antibodies were used: phycoerythrin (PE) conjugated rat anti-mouse CD4, CyChrome (CyC) conjugated rat anti-mouse CD8 and FITC conjugated rat anti-mouse CD3, all purchased from BD Pharmingen, CA. Samples were measured and analysed in a FACSCalibur flow-cytometer (Becton Dickinson, San Jose, CA), using the CellQuest software. Generally 10.000 events were recorded. Thymocytes were gated according to their size and granularity on forward and side scatter dot plots. The gate set on untreated control living thymocytes was used for the analysis of all samples. We used fluorescent dot plots for both comparing the different samples and for calculating the ratio of positively stained cells.

### Immunofluorescence

Frozen thymic sections (7–10 μm thick) were fixed in cold acetone, then air-dried and blocked using 5% bovine serum albumin (BSA) in PBS for 20 min before staining with a-Ly51-PE (clone 6C3, BD Biosciences) and a-EpCAM1-FITC (clone G8.8, hybridoma from the Dept. Immunology and Biotechnology, University of Pecs) antibodies for at least 30 minutes to visualise the thymic epithelial network (EpCAM-1 is a marker for all TECs, but stains the medullar area stronger than the cortical area, while Ly51 stains only cortical TECs). The remaining sections were stained under the same conditions with a-CD4-FITC (YTS191.1, hybridoma from the Dept. Immunology and Biotechnology, University of Pecs) and a-CD8-Alexa fluor 647 (clone 53–6.7, BD Biosciences) to analyse the staining pattern of the thymocytes. The sections were analysed using an Olympus BX61 microscope equipped with CCD-camera and AnalySIS software.

### Electron microscopy

In order to investigate ultra-structural changes of the thymic lobes, mice were sacrificed after three days of treatment and their thymi were fixed in buffered 2.5% formaldehyde―2.5% glutaraldehyde solution for 24 hours at 4°C. After washing in phosphate buffer, the samples were fixed in 1% osmium tetroxide in 0.1 M PBS for 35 minutes. After that, the samples were washed in buffer several times for 10 minutes and dehydrated in an ascending ethanol series, including a step of uranyl acetate (1%) solution in 70% ethanol to increase contrast. Dehydrated blocks were transferred to propylene oxide before being placed into aluminium-foil boats containing Durcupan resin (Sigma) and then embedded in gelatine capsules containing Durcupan. The blocks were placed in thermostate for 48 hours at 56°C. From the embedded blocks 1 μm semi-thin and serial ultrathin sections (70 nanometer) were cut with Leica ultramicrotome, and mounted either on mesh, or on Collodion-coated (Parlodion, Electron Microscopy Sciences, Fort Washington, PA) single-slot copper grids. Additional contrast was provided to these sections with uranyl acetate and lead citrate solutions, and they were examined with JEOL 1200EX-II electron microscope.

### Immunoblot analysis

Tissue samples were taken from animals sacrificed after three or seven days of treatment. The thymi of the mice were carefully dissected and 25 mg tissues were homogenised in ice-cold 10 mM Tris buffer, pH 7.4 (containing 0.5 mM sodium metavanadate, 1mM EDTA and protease inhibitor cocktail (1:200); all purchased from Sigma-Aldrich, Steinheim, Germany). Homogenates (10 μg each) were boiled and subjected to 10% sodium dodecyl sulphate polyacrylamide gel electrophoresis then were transferred to nitrocellulose membranes. The membranes were blocked in 5% low fat milk for 1.5 h at room temperature before then being exposed to primary antibodies at 4°C overnight in a blocking solution. The following antibodies were used: Polyclonal caspase 3 (1:500, clone H-277), monoclonal Histone H1(1:200), polyclonal Cytochrome c, polyclonal AIF, monoclonal phospho-p38 MAPK (Thr180/Tyr182), polyclonal phospho-extracellular signal-regulated kinase (ERK)1/2 (Thr202/Tyr204), polyclonal phospho-specific c-Jun N-terminal kinase (JNK) Thr183-Tyr185, polyclonal Bad (D24A9), polyclonal phospho-Bad (Ser112), polyclonal BIM and polyclonal Bax antibodies (each 1:500 dilution), monoclonal Glyceraldehyde-3-phosphate dehydrogenase (1:2000, clone 6C5). Antibodies were purchased from Cell Signaling Technology (Beverly, MA, USA) except from caspase 3, Histone H1 that were bought from Santa Cruz Biotechnology (Wembley, UK), Glyceraldehyde-3-phosphate dehydrogenase antibody was obtained from Merck Millipore. Appropriate horseradish peroxidase-conjugated secondary antibodies were used at a 1:5.000 dilution (anti-mouse and anti-rabbit IgGs; Sigma-Aldrich, Steinheim, Germany) and visualised by enhanced chemiluminescence (Amersham Biosciences, Piscataway, New Jersey, USA). The films were scanned, and the pixel volumes of the bands were determined using NIH Image J software (Bethesda, Maryland, USA). For membrane stripping and re-probing, the membranes were washed in a stripping buffer (0.1 M glycine, 5 M MgCl_2_, pH 2.8) for an hour at room temperature. After washing and blocking, the membranes were incubated with primary antibodies for non-phosphorylated or loading control proteins.

### RNA isolation and quantitative polymerase chain reaction (qPCR) analysis

To investigate the relative expression of thymic epithelial cell markers major histocompatibility complex (MHC)II and autoimmune regulator (AIRE), we used a real-time qPCR approach after seven days of treatment. From carefully dissected thymi, the total RNA was extracted and DNase digestion was performed using the NucleoSpin RNA isolation kit (Macherey-Nagel, Düren, Germany) as described in the manufacturer’s manual. For quality control, RNA purity was verified using the optical density (OD)_260/280_ ratio and was found to be between 1.8 and 2.0. The total RNA (1.0μg) was reverse-transcribed to cDNA using the High-Capacity cDNA reverse transcription kit (Applied Biosystems). Subsequent qPCR reactions for MHCII and AIRE were performed in duplicate on the Applied Biosystems HT7500 system using the Absolute QPCR SYBR Green low ROX Mix (Abgene, Epsom, UK) with specific primers (MHC II forward primer: 5’-CTA GCC AAG TCC CTC CTA AGG-3’, reverse primer: 5’- ATC TCA GAC TGA TCC TGG CAT -3’; AIRE forward primer: 5’-ACC TAA ACC AGT CCC GGA AAG-3‘, reverse primer: 5’-CGA GGC TCC AGT GCT T-3’). The three step qPCR was performed using 58°C annealing temperature and 30sec elongation period. Analysing melting curves validated the specificity of products from each primer set. All experimental samples were analysed and normalised with the internal control gene, 18 S rRNA (forward primer: 5’-GGG TCG GGA GTG GGT AAT TT-3’, reverse primer: 5’-AGA AAC GGC TAC CAC ATC CAA-3’). Relative quantification of the fold-change was performed comparing *Ct* values from individual mice, applying the 2^-ΔΔCt^ method [22,23].

### Statistical analysis

All experiments were repeated at least three times, including at least three animals in each group, for each experiment. Accordingly, the mean + the standard error of the mean (SEM) values of the individual animals (n≥9) were presented in the figures and throughout the text. On the other hand, for comparing groups, we utilised data of the experiments repeated at least three times, including at least three animals in each groups. We used one-way or two-way analysis of variance, followed by Tukey’s post-hoc test. When the F-test indicated unequal variances, the Kruskal-Wallis test was performed. Differences were considered significant at values of p<0.05 or lower.

## Results

### Cuprizone induced acute thymic atrophy

**Fig 1:**
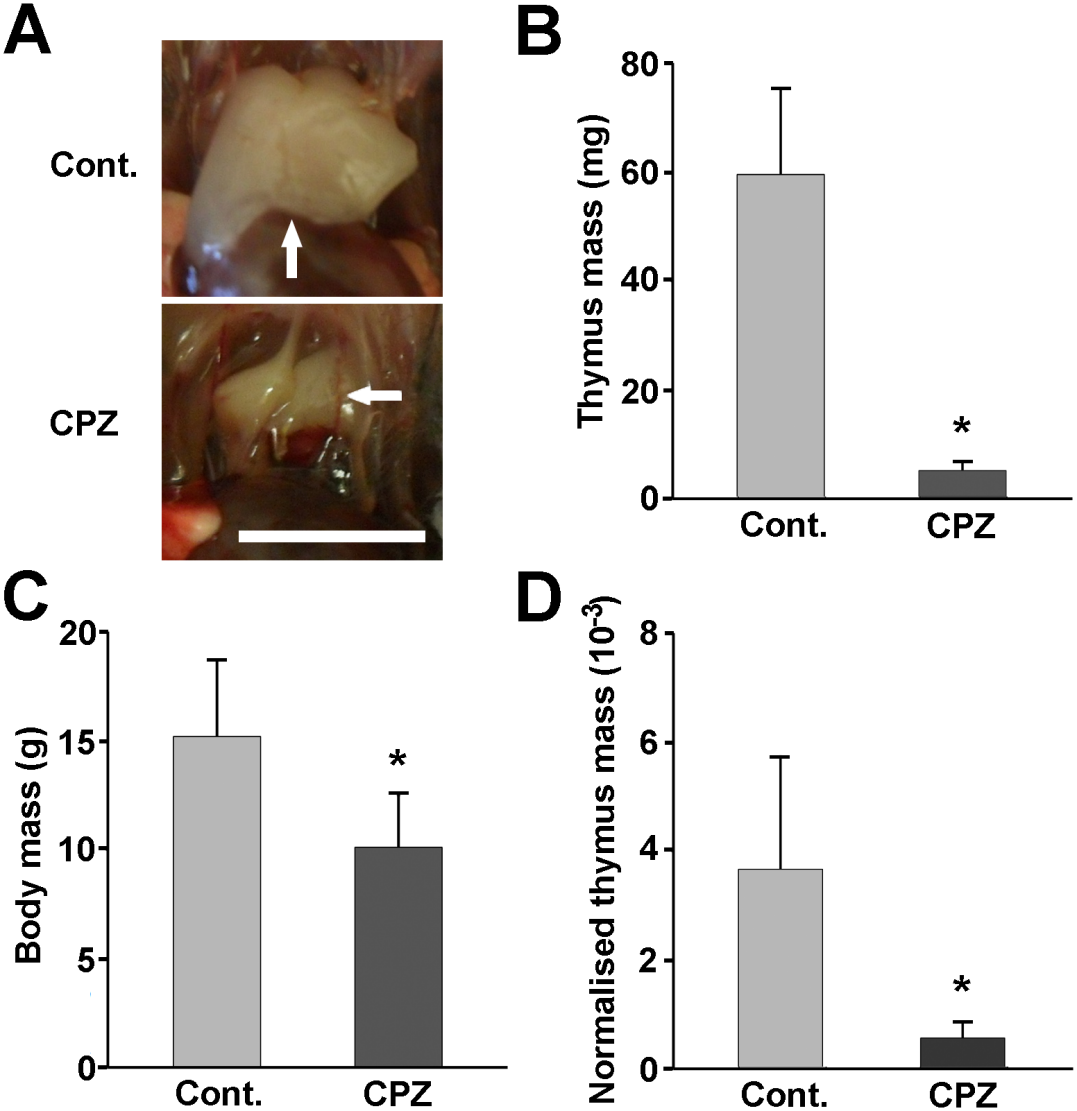
Macroscopic changes of the thymi upon cuprizone treatment. Four week-old male mice were treated with cuprizone for one week. Representative photographs (A) of the open chest of a control (Cont.) and a cuprizone-treated (CPZ) animal are demonstrated. Arrows point to the thymi of the mice. The scale bar indicates 5 mm. Thymus mass (B), body mass (C) and relative thymus mass (thymus tissue mass/ body mass) (E) of control (grey bars) and cuprizone-treated (black bars) animals are presented as bar diagrams, mean + SEM (n≥9). * denotes a significant difference from control p<0.05.

In order to investigate the effect of cuprizone on the thymus, four-week-old male C57BL/6 mice were fed with pulverised chow containing 0.2% of the drug. As early as after one week of cuprizone administration, severe thymic atrophy was observed (Fig 1) that was accompanied by significant thymic tissue mass loss when compared to the control (Fig 1B). Since cuprizone treatment resulted in a significant weight loss for the animals as well (Fig 1C), we normalised their thymic mass to their body mass, and found this relative thymus mass still reduced in the cuprizone-treated mice (Fig 1D), indicating a disproportional thymus involution in these animals. The absolute thymocyte number was also found to be lowered from 1.7±0.2 x10^8^ to 7.8±2.3 x10^6^ upon cuprizone treatment.

### Cuprizone-induced cell death was predominantly apoptotic

**Fig 2:**
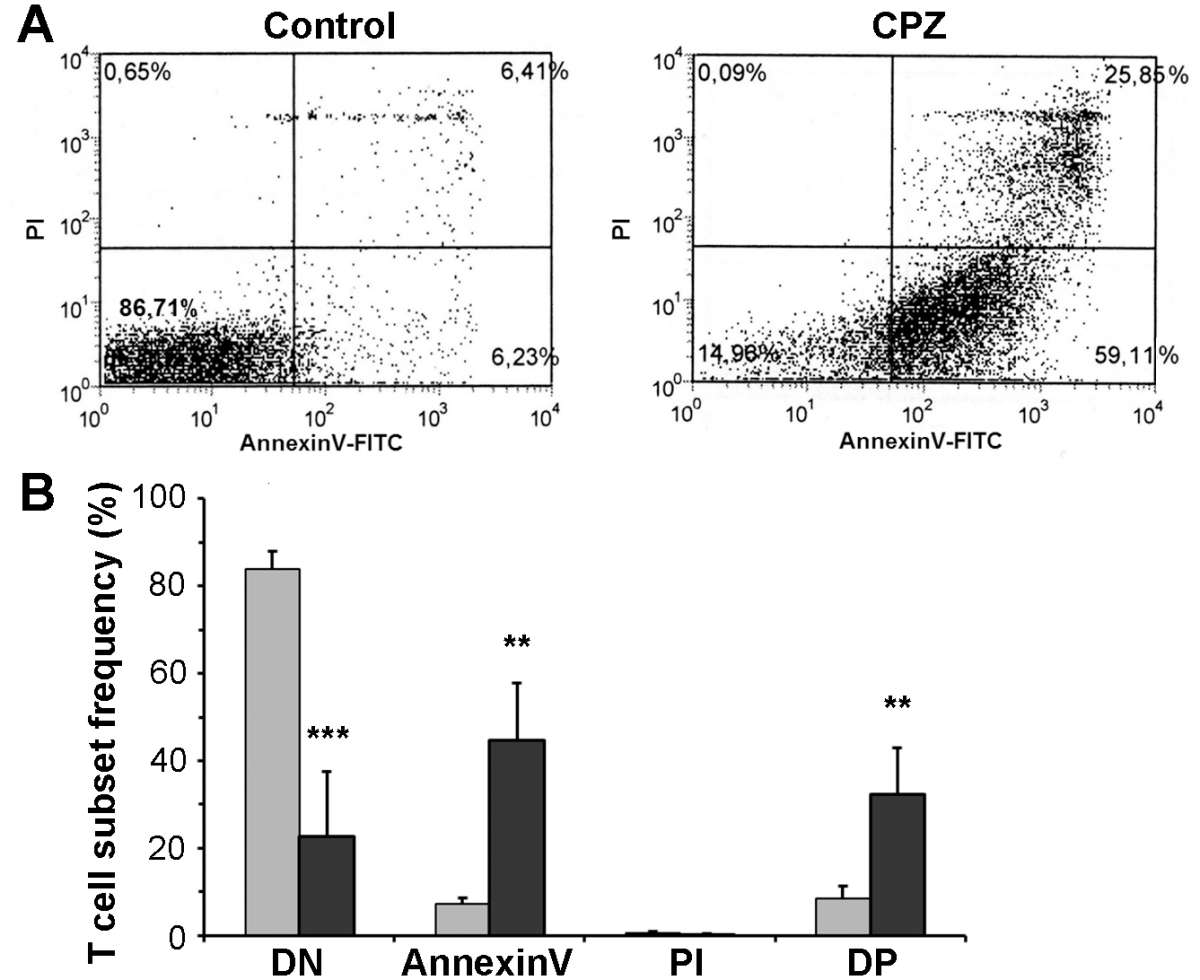
Characterisation of cuprizone-induced cell death. Four week-old male mice were treated with cuprizone for one week. Type of cell death was determined using flow cytometry following double staining with FITC-labelled AnnexinV and propidium iodide thymus suspensions of untreated (Control, grey bars) and cuprizone-treated (CPZ, black bars) mice. Results are presented as representative dot-plots (A) and bar diagrams (B), mean + SEM (n≥9). Significant difference from control; **p<0.01, *** p<0.001. DN: live cells (lower left quadrant); AnnexinV: early apoptotic cells (lower right quadrant); PI: necrotic cells (upper left quadrant); DP: late apoptotic cells (upper right quadrant).

We investigated the type of cell death responsible for the substantial cuprizone-induced thymic involution by performing flow-cytometry on the thymic cell suspension following double staining the cells with FITC-conjugated Annexin V and propidium iodide. Propidium iodide is excluded by viable cells. On the other hand, it can penetrate cell membranes of dying or dead cells, and intercalate into double-stranded nucleic acids, thereby increasing the intensity of its fluorescence manyfold. Annexin V binds to phosphatidylserine exposed on the surface of apoptotic cells only, and therefore, this staining technique can differentiate between living (double negative), early apoptotic (Annexin V single positive), and late apoptotic/necrotic (AnnexinV and propidium iodide double positive) cells. As we found, in the untreated control mice, most of the cells were double negative (83.9 ± 4.1%), which was substantially decreased in cuprizone treated animals (22.8 ± 13.2%, p<0.001, Fig 2). In these mice, the decrease in the living cells was accompanied by a significant increase of the early apoptotic (44.6 ± 13.2%, p<0.001) and late apoptotic/necrotic cells (32.4 ± 10.5%, p<0.01, respectively Fig 2). We could not observe propidium iodide single positivity among the animals regardless of the treatment. All these data indicate that the cuprizone-induced death was preponderantly apoptotic.

### Cuprizone-induced cell loss in the cortex was more severe than in the medulla

**Fig 3:**
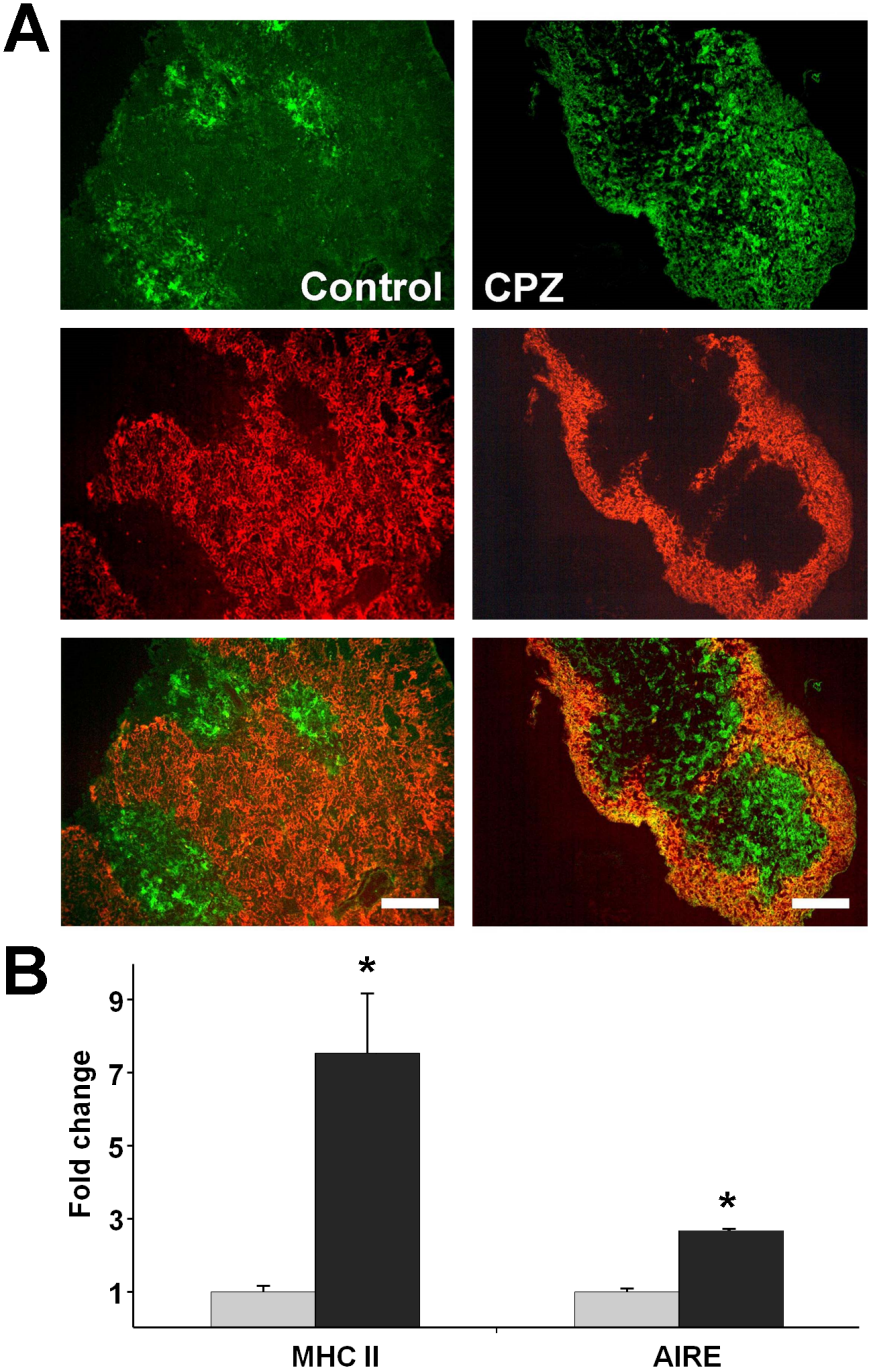
Effect of cuprizone on thymic epithelial cells. Four week-old male mice were treated with cuprizone for one week, then immune-staining (A) with FITC-labelled anti-EpCAM1 (green) and PE-labelled anti-Ly-51 (red) antibodies was performed on thymic sections of untreated (Control) and cuprizone-treated (CPZ) mice. Representative images (A) are presented of the green channel (top panels), the red channel (middle panels) and the merged channels (bottom panels) of three independent experiments, including at least three animals in each group for each experiment. Fluorescent photographs were taken using a 10x objective. The scale bar indicates 200 μm. In a parallel experiment, thymic MHC II and AIRE mRNA expression (B) was determined by using qPCR analysis in untreated (grey bars) and cuprizone-treated (black bars) mice. Results are presented as fold change, mean + SEM (n≥9). Significant difference from control; *p<0.05.

We determined macroscopic morphological characteristics of the cuprizone-induced thymus involution by fluorescent microscopy after double staining thymus sections with FITC-labelled anti-EpCAM1 and PE-labelled anti-Ly-51 antibodies. The former antibody stains mostly the medulla while the latter stains the cortex of the thymus. We observed more substantial cell loss in the cortex than in the medulla (Fig 3A). To support these findings, we determined the MHCII and AIRE mRNA levels in the thymi of the control and cuprizone treated mice. We found a significant abundance of both of these medulla-associated markers in the cuprizone treated animals (Fig 3B), indicating that cuprizone preferentially affected cortical cells.

### Cuprizone eliminated mostly the CD4-CD8 double positive immature thymocytes

**Fig 4:**
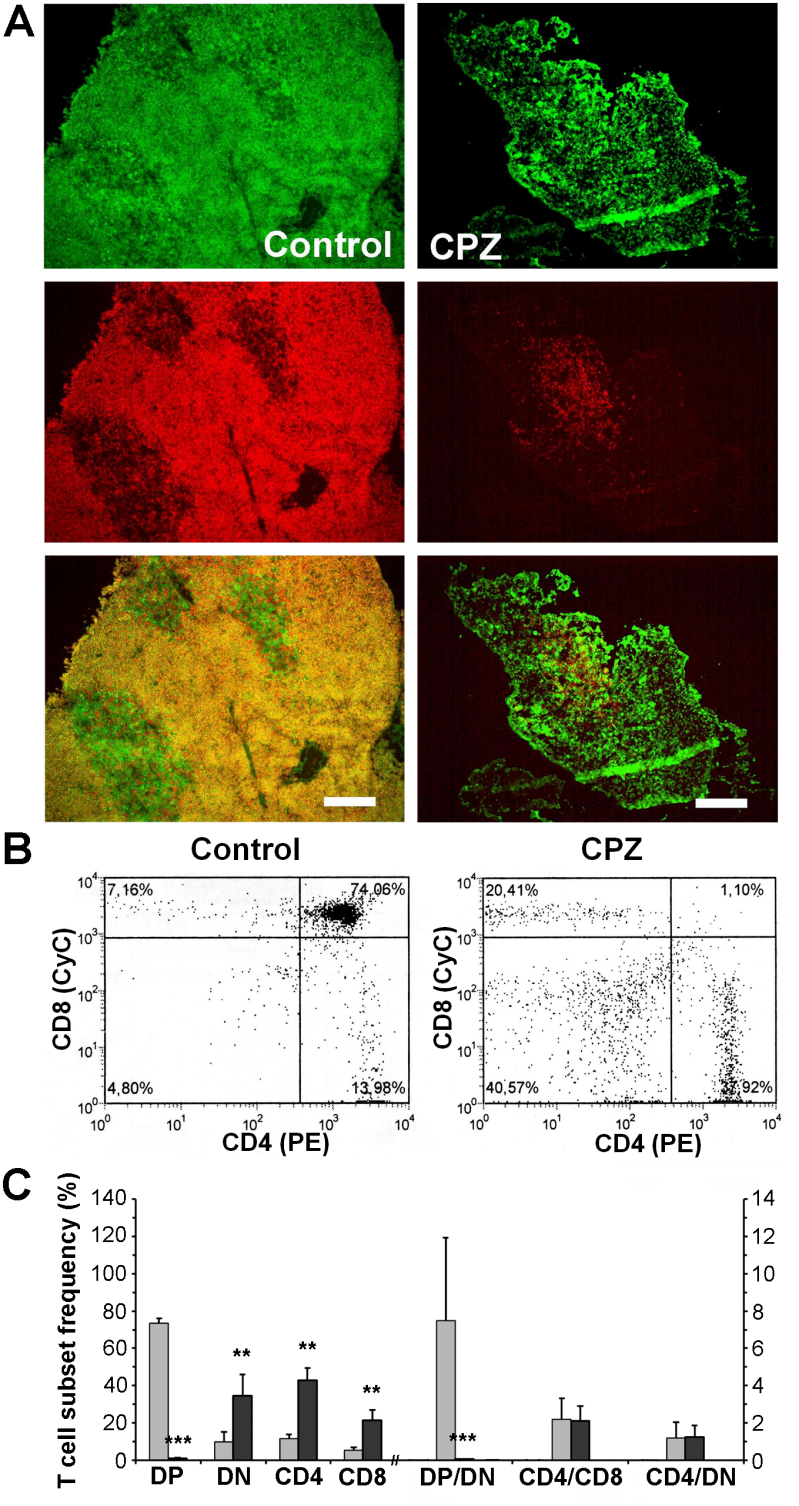
Effect of cuprizone treatment on thymocytes. Four week-old male mice were treated with cuprizone for one week, then immune-staining (A) with FITC-labelled anti-CD4 (green) and Alexa647-labelled anti-CD8 (red) antibodies was performed on thymic sections of untreated (Control) and cuprizone-treated (CPZ) mice. Representative images (A) are presented of the green channel (top panels), the red channel (middle panels) and the merged channels (bottom panels) of three independent experiments including at least three animals in each group for each experiment. Fluorescent photographs were taken using a 10x objective. The scale bar indicates 200 μm. In a parallel experiment, flow cytometry was performed on thymus suspensions of untreated (Control, grey bars) and cuprizone-treated (CPZ. black bars) mice following double staining with PE-labelled CD4 and CyChrome (CyC)-labelled CD8 antibodies. Results are presented as representative dot-plots (B) and bar diagrams (C), mean + SEM (n≥9). Significant difference from control; **p<0.01, *** p<0.001. DN: CD4^-^/CD8^-^ cells (lower left quadrant); CD4: CD4^+^ cells (lower right quadrant); CD8: CD8^+^ cells (upper left quadrant); DP: CD4^+^/CD8^+^ cells (upper right quadrant); DP/DN: ratio of DP and DN cells; CD4/CD8: ratio of CD4^+^ and CD8^+^ cells; CD4/DN: ratio of CD4^+^ and DN cells. Please note that the x-axis is broken, and the ratios are measured on the right y-axis.

To further analyse cuprizone’s effect on the thymus, we performed anti-CD4 (green) and anti-CD8 (red) immunofluorescence microscopies. On merged images of the control thymi, the cortex appeared yellow as it is mainly occupied by CD4^+^CD8^+^ thymocytes while the medulla appeared green because of the predominance of CD4^+^ cells (Fig 4A). Cuprizone-treatment resulted in an almost complete disappearance of the double positive and a relative increase of the CD4^+^ areas, as well as an overall less dense staining of the shrunken thymi (Fig 4A). To ensure that the double positivity of the control cortices indeed resulted mainly from the presence of the CD4^+^CD8^+^ thymocytes, we performed anti-CD4 and anti-CD8 flow-cytometry on the thymic suspensions. To exclude dead or apoptotic cells, the gate was set on untreated control thymocytes and was fixed for the whole analysis. In the case of the four week-old male C57BL/6 control mice, we found that the most considerable population was that of CD4^+^CD8^+^ thymocytes (73.4 ± 2.6%, Fig 4B and 4C). The most immature CD4^-^CD8^-^ thymocytes made up 9.8 ± 5.3%, while the most mature CD4^+^ and CD8^+^ thymocytes represented 11.6 ± 2.2 and 5.3 ± 1.7% of the total population, respectively (Fig 4B and 4C). One week of cuprizone treatment completely eradicated CD4^+^CD8^+^ thymocytes (1.0 ± 0.1%, p<0.001, Fig 4B and 4C). If we consider the total cell numbers of the thymi, this decrease was from 1.3 ± 0.2 x10^8^ to 8.1 ± 3.5 x10^4^ double positive thymocytes suggesting a complete disappearance of this cell population. This finding is reflected by a decrease of the double positive/double negative thymocyte ratio from 7.5 ± 4.5 to 0.04 ± 0.02 (p<0.001) upon cuprizone treatment, while all other ratios (CD4^+^/CD8^+^ and CD4^+^/double negative) remained practically unchanged (Fig 4C).

**Fig 5:**
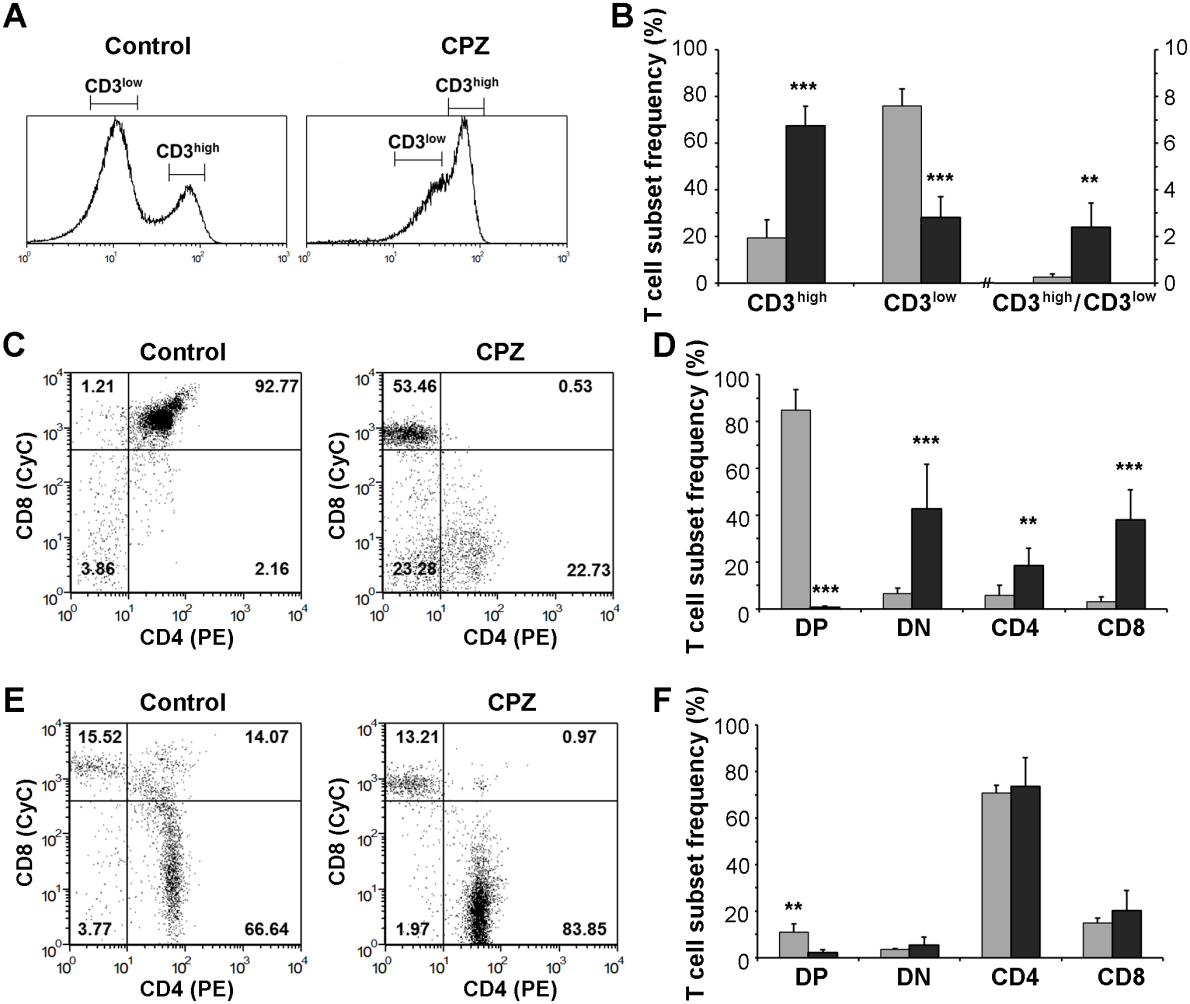
Effect of cuprizone treatment on thymocyte subpopulations. Four week-old male mice were treated with cuprizone for one week, then flow cytometry was performed on thymus suspensions on thymus suspensions of untreated (Control, grey bars) and cuprizone-treated (CPZ, black bars) mice following triple staining with FITC-labelled CD3, PE-labelled CD4 and CyChrome (CyC)-labelled CD8 antibodies. Based on CD3 positivity (A, B) the data on CD4 and CD8 were allocated to low (CD3^low^; C, D) and high (CD3^high^; E, F) CD3 positivity subgroups (the ranges are indicated in A). Results are presented as representative line charts (A), dot-plots (C, E) and bar diagrams (B, D, F), mean + SEM (n≥9). Significant difference from control; **p<0.01, *** p<0.001. DN: CD4^-^/CD8^-^ cells (lower left quadrant); CD4: CD4^+^ cells (lower right quadrant); CD8: CD8^+^ cells (upper left quadrant); DP: CD4^+^/CD8^+^ cells (upper right quadrant); CD3^high^/CD3^low^: ratio of CD3^high^ and CD3^low^ cells. Please note that the x-axis in B is broken, and the ratio is measured on the right y-axis.

CD3 expression increases along thymocyte maturation. Accordingly, we stained thymus suspensions for CD3, performed flow-cytometry, and assessed the ratio of immature (CD3^low^) and mature (CD3^high^) thymocytes in the untreated and cuprizone-treated groups. In four week-old male C57BL/6 control mice, we found that the CD3^low^ and CD3^high^ thymocytes comprised about 76 and 19%, respectively, of the whole thymocyte population. One week of cuprizone treatment resulted in a significantly lower proportion of immature thymocytes (28.2 ± 8.8, p<0.001) and a corresponding increase in the ratios of mature (67.5 ± 8.5, p<0.001) thymocytes (Fig 5A and 5B). Thereby, cuprizone treatment increased the CD3^high^/CD3^low^ ratio from about 0.25 to 2.4 (p<0.001, Fig 5B) indicating that cuprizone eliminated immature thymocytes preferentially. Additionally, we triple stained thymocytes of control and cuprizone-treated animals for CD3, CD4 and CD8, and performed flow cytometry on them. When we filtered the results for the CD3^low^ (Fig 5C and 5D) and CD3^high^ (Fig 5E and 5F) subpopulations, we found changes in the T-cell subset frequencies upon cuprizone treatment that were completely consistent with those we found on the unfiltered population, as well as being consistent with our present knowledge of CD3, CD4 and CD8 expression during thymocyte maturation. Namely, almost all CD4-CD8 double positive cells were CD3^low^ and were almost completely depleted by cuprizone (Fig 5C and 5D). Additionally, there was CD8 and CD4 dominance in the CD3^low^ and CD3^high^ subset, respectively that was augmented by the cuprizone treatment (Fig 5C–5F).

### Cuprizone induces subcellular structural alterations in thymic cells

**Fig 6:**
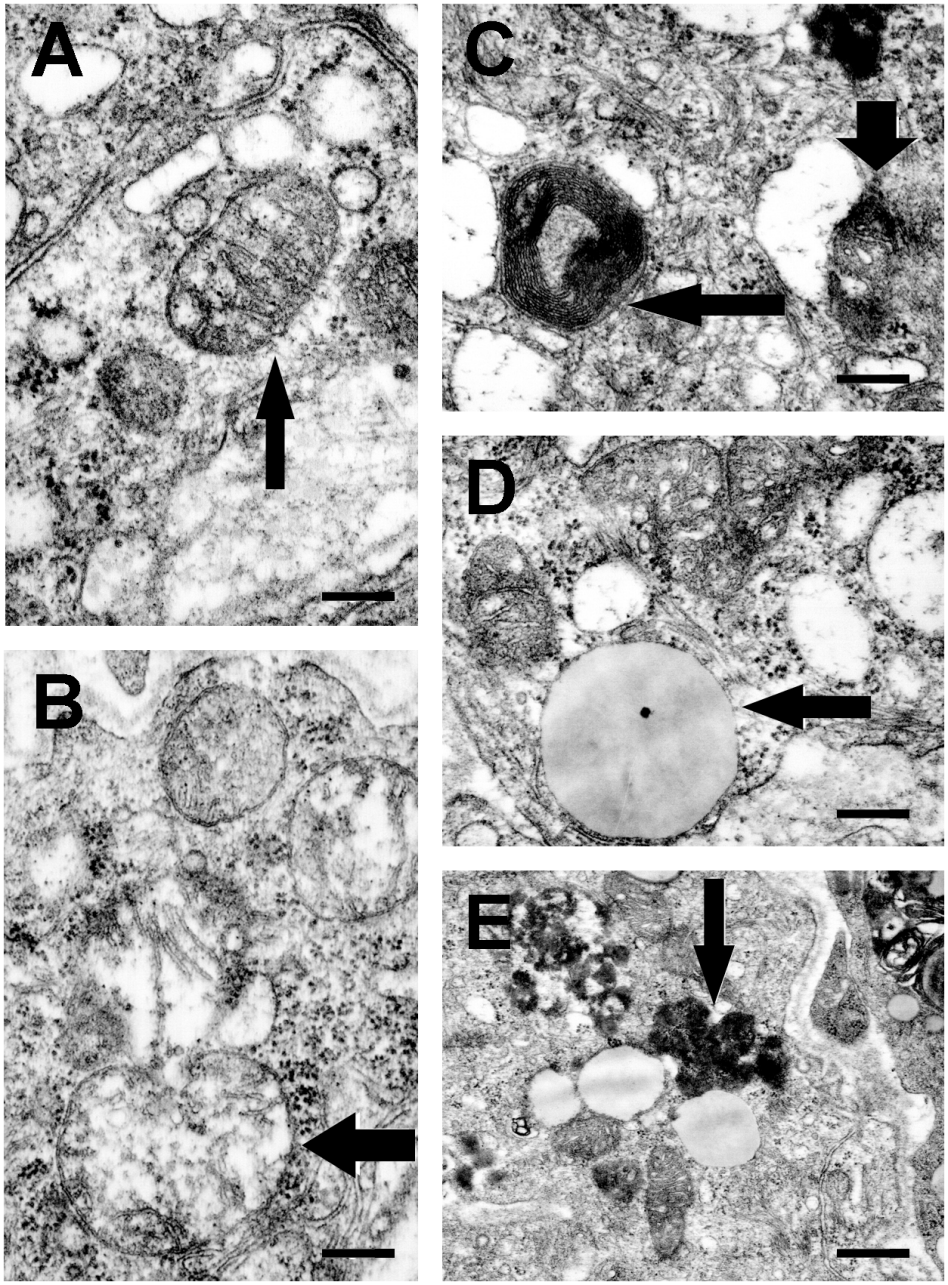
Effect of cuprizone treatment on subcellular morphology. Four week-old male mice were treated with cuprizone for one week. Subcellular morphology was assessed in ultrathin thymic sections of control (A) and cuprizone-treated (B-E) mice. Representative images are presented of three independent experiments including at least three animals in each group for each experiment. Arrows indicate normal (A) and enlarged (B) mitochondria, a large lipid droplet (D), and large lysosomes packed with darkly stained material (E). Horizontal thin and vertical thick arrows in (C) point to myelin body and a degraded mitochondrion, respectively. Scale bars indicate 200 nm.

Cuprizone was reported to induce giant mitochondria formation and mitochondrial malfunctioning in mouse liver [24]. Since mitochondria are major regulators of the cell death process [25], we assessed cuprizone’s effect on thymic mitochondria by using electron microscopy. As demonstrated in Fig 6, cuprizone-treated thymic cells contained both enlarged and medium-sized mitochondria (Fig 6B); the latter are similar to those observed in untreated animals (Fig 6A). The diameter of the enlarged mitochondria did not reach 1 μm, the conventional threshold to be categorised as mega-mitochondria [26]. In addition to enlarged mitochondria, cuprozine treatment resulted in the degradation of cellular organelles, such as mitochondria (Fig 6C). Myelin-bodies (Fig 6C), large lipid droplets (Fig 6D) and large lysosomes packed with dark-staining material (Fig 6E) were also frequently observed.

### Cuprizone-treatment activated mitochondrial death pathways

To investigate which death pathways were involved in cuprizone-induced thymic atrophy, we examined caspase activation, pro-apoptotic mitochondrial inter-membrane protein release, and major pro- and anti-apoptotic B cell lymphoma (BCL) proteins by using immunoblot analysis of thymus homogenates of untreated and cuprizone-treated animals. We used thymi after only three days of cuprizone-feeding since we were interested in the processes leading to the massive thymocyte loss.

**Fig 7:**
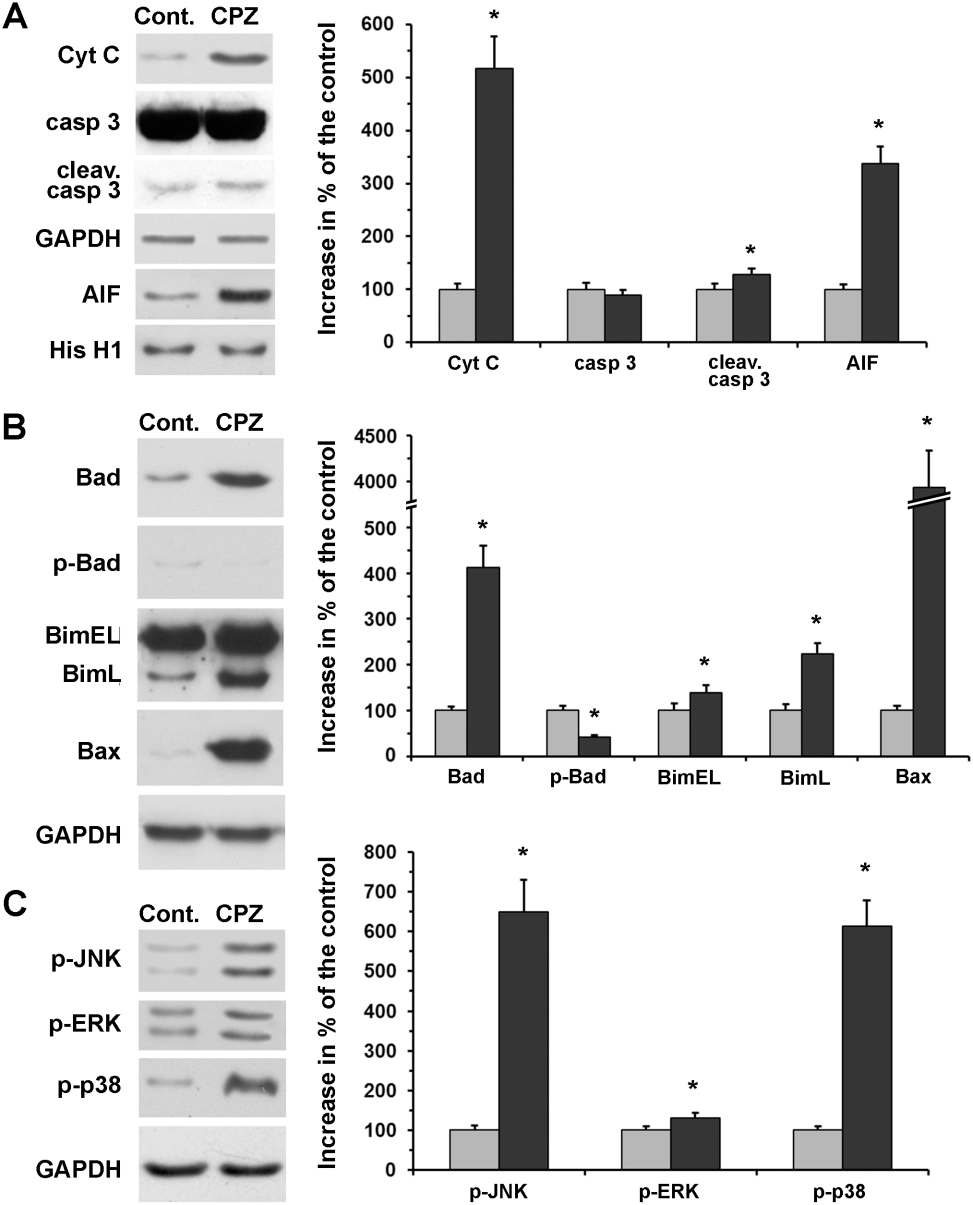
Effect of cuprizone treatment on death pathway and signalling proteins in the thymus. Four week-old male mice were treated with cuprizone for three days. Steady-state cytoplasmic levels of cytochrome C (Cyt C) (A), caspase 3 (casp 3) (A), cleaved caspase 3 (cleav. casp 3) (A) and nuclear apoptosis inducing factor (AIF) content (A), as well as cellular levels of Bad (B), BimEL (B), BimL (B) and Bax (B) were assessed in the thymi of untreated (Cont., grey bars) and cuprizone-treated (CPZ, black bars) mice by using specific primary antibodies and immunoblotting. The activation state of Bad (p-Bad) (B), JNK (p-JNK) (C), ERK (p-ERK) (C) and p38 MAPK (p-p38) (C) was also determined by using phosphorylation-specific primary antibodies and immunoblotting. GAPDH (A-C) and histone H1 (His H1) (A) were used as loading controls for cytoplasmic/cellular and nuclear fractions, respectively. Results are presented as representative immunoblots and bar diagrams, mean + SEM (n≥9). Significant difference from control; *p<0.05. Please note that the y-axis in B is broken to accommodate the very high Bax value.

We detected a substantial release to the cytoplasm of cytochrome C, in addition to a resulting increased cleavage i.e. activation of caspase 3 (Fig 7A), but not of caspase 8 (data not shown). We also observed nuclear translocation of AIF (Fig 7A), indicating that the cuprizone-induced apoptosis was of mainly mitochondrial origin. We found that the pro-apoptotic Bcl-2 family members Bim, Bax and Bad had overlapping functions in cuprizone-induced killing of CD4-CD8 double-positive thymocytes. The expression level of all three proteins was increased significantly after three days of cuprizone-treatment, although, Bax expression was augmented at least 10 times over the other two (Fig 7B). Additionally, increased Bad expression was accompanied by decreased phosphorylation of the protein (Fig 7B) that emphasises its pro-apoptotic role in cuprizone-induced thymocyte loss.

### Cuprizone-treatment activated all three MAP kinases

A number of reports implicate MAPK activation as a causative factor of mitochondrial damage and apoptotic cell death [27,28]. Therefore, we assessed phosphorylation i.e. activation of JNK, ERK and p38 in thymus homogenates by using phosphorylation-specific primary antibodies and immunoblotting. After three days of cuprizone-feeding we found increased activation of all three MAP kinases. JNK and p38 phosphorylation were increased upon cuprizone treatment by over 600% while ERK phosphorylation increased by only about 30% (Fig 7C).

## Discussion

To enhance our knowledge of the degenerative animal model of MS, we investigated the effect of cuprizone on the thymus, the site of T-cell maturation and selection. Although, thymus involution in the cuprizone model is extremely apparent (Fig 1A), we could not find any previous report mentioning it. Cuprizone-treatment results in reduced thriving of the animals [29]; however, thymus retardation upon cuprizone-treatment was more pronounced than that of the rest of the body (Fig 1D), resulting mainly from apoptotic death (Fig 2).

By using immunostaining and fluorescent microscopy, we demonstrated that cuprizone-treatment affected the cortex over the medulla (Fig 3A), supported by the relatively higher expression level (Fig 3B) of AIRE, a gene expressed by the thymic medullary epithelial cells [30], in the thymi of cuprizone-treated mice. Since the cortex is associated mostly with the positive selection of T-cells while the medulla is the location of the negative selection of auto-reactive T-cells from the mature repertoire, this result indicated that cuprizone affected immature T-cells more severely than it affected the mature ones. Accordingly, the observed relatively higher expression level of MHCII (Fig 3B) in the thymi of cuprizone-treated mice resulted mainly from the preservation of mature T-cells residing dominantly in the medulla.

We intended to identify the T-cell subpopulation that was most sensitive to cuprizone. Immunofluorescence and flow-cytometry analysis revealed that cuprizone eradicated CD4^+^CD8^+^ T-cells while practically did not affect ratios of other T cell subpopulations (Fig 4). These results were confirmed when we filtered the results of CD4 and CD8 positivity of the cells according to their CD3 status. The double positive subset of T-cells of low CD3 status was abolished by cuprizone while there was CD8^+^ dominance among the surviving cells, and a corresponding CD4^+^ dominance was observed among the cuprizone-resistant T-cells of high CD3 status (Fig 5). These data are in accord with our aforementioned findings and the present view of T-cell maturation. Namely, immature double negative T-cells first express CD8 before becoming double positive, which cells undergo positive and negative selection to reach their mature CD4^+^ or CD8^+^ phenotype, while CD3 positivity increases along the maturation process.

By chelating copper, cuprizone may interfere with cellular energy metabolism. Supporting this notion, megamitochondrion formation was observed as early as after three days of cuprizone-feeding in mouse liver [24,29], and the oligodendrocytes of the corpus callosum [31]. Furthermore, increased ROS production, and decreased activities of the various complexes of the respiratory chain were found in the mitochondria of cuprizone-treated oligodendroglia cells [32,33]. We found degraded, as well as enlarged, although not giant, mitochondria in the thymi of cuprizone-treated mice (Fig 6). Additionally, myelin-bodies, large lipid droplets, and large lysosomes were also frequently present in these cells (Fig 6). The observed large lysosomes could result from lysosome membrane permeabilisation, which contributes to thymocyte apoptosis by releasing lysosomal proteases, thereby causing the degradation of vital cytosolic proteins and the activation of additional hydrolases, including caspases [34]. This scenario is consistent with the finding that, unlike in the case of oligodendrocytes [6], both AIF- and caspase-mediated apoptosis was observed in the thymi of cuprizone-treated mice (Fig 7A). On the other hand, cytoplasmic translocation of mitochondrial cytochrome C (Fig 7A) may entirely account for the observed caspase-3 activation.

Both nuclear translocation of AIF and cytoplasmic translocation of cytochrome C could result from mitochondrial outer membrane permeability, which is regulated by pro-apoptotic members of the BCL-2 family [35]. Bad forms heterodimers with other family members or BH3-only proteins such as Bax and Bim, attach to the outer mitochondrial membrane, and destabilize it [35]. Bim, an essential mediator of apoptosis in thymocytes, has three isoforms (BimS, BimL and BimEL), which promote intrinsic apoptosis to different extents [36]. Accordingly, we detected enhanced expression of Bad, Bax and Bim upon cuprizone treatment (Fig 7B). Activation of ERK1/2 was reported to promote phosphorylation of BimEL targeting it to proteosomal degradation [37]. ERK1/2 was also indicated in Bad phosphorylation, preventing its association with other pro-apoptotic BCL-2 proteins [38]. In contrast, JNK and p38 MAPK activation was found to trigger Bad and Bim expression, labilise mitochondrial integrity, induce ROS production and promote apoptosis [39]. Accordingly, we found that all three MAPK families become activated upon cuprizone treatment; however, JNK and p38 activation was overwhelmingly more pronounced than was that of ERK1/2 (Fig 7C).

During development, thymocytes undergo positive and negative selection, eliminating roughly 98% of CD4^+^CD8^+^ cells before they maturate along the CD4^+^ or CD8^+^ phenotype. Additionally, when the thymus undergoes physiologic or pathologic involution during aging, infectious diseases, sepsis, malnutrition, physical or emotional stress, chemotherapeutics, glucocorticoids or radiation injury, cortical double positive cells, primarily, suffer apoptosis, while CD4^+^ or CD8^+^ cells are less affected [40–43], indicating that cortical double positive cells represent the most vulnerable cell population in the thymus. In this respect, cuprizone-induced thymus involution is similar to all aforementioned conditions. In other respects, the cuprizone model shows both similarities and differences compared to physiological or steroid-induced thymic aging.

The thymus undergoes adiposeous involution during aging. We observed large lipid droplets (Fig 6D) similar to those found in physiological and steroid-induced accelerated aging [41]. However, the thymic epithelium is affected differently by these conditions. The medullary region suffers less damage compared to the cortical region in the cuprizone model, which is in marked contrast to physiological or steroid-induced aging [44,45]. The observed enlarged lysosomes (Fig 6E) could be involved not only in apoptosis but also in other types of cell death. There is increasing evidence that the lysosome is also involved in the pathogenesis of a variety of neurodegenerative diseases, including Alzheimer's disease, Parkinson's disease, Huntington's disease, and amyotrophic lateral sclerosis [34].

The thymus can have dual functions in MS. It can be the organ, where potentially self-reactive T-cells mature and differentiate inducing or exacerbating the disease. However, it is also the cradle of regulatory T-cells that can potentially suppress self-reactive immune responses, locally [46,47] Our results do not indicate a functional relationship between cuprizone-induced thymus involution and the absence of inflammatory responses or the selective demyelination observed in the cuprizone model. Rather, cuprizone-induced thymocyte and oligodendrocyte apoptosis seems to occur parallel to each other, and in both cases the toxin affects the most vulnerable cells in the given organ. It raises the possibility that similar selective elimination of the most vulnerable cell type in other organs is responsible for the absence of thriving that is characteristic of the cuprizone model. An important feature of the cuprizone model is that after termination of the toxin feeding, an accelerated thriving and regeneration occurs. Therefore, the cuprizone model could be valuable in studying thymus regeneration as well as the remyelination processes.
